# Resveratrol therapy improves liver function via estrogen-receptors after hemorrhagic shock in rats

**DOI:** 10.1371/journal.pone.0275632

**Published:** 2022-10-13

**Authors:** Alexander Wolf, Tobias Fink, Jochen Hinkelbein, Thilo Mertke, Thomas Volk, Alexander Mathes

**Affiliations:** 1 Department of Anesthesiology, Critical Care and Pain Medicine, Universitätsklinikum Knappschaftskrankenhaus Bochum GmbH, Bochum, Germany; 2 Department of Anesthesiology, Critical Care and Pain Therapy, Saarland University Hospital, Homburg (Saar), Germany; 3 Department of Anesthesiology and Intensive Care Medicine, University Hospital and Medical Faculty, University Hospital Cologne, Cologne, Germany; Texas A&M University, UNITED STATES

## Abstract

**Background:**

Resveratrol may improve organ dysfunction after experimental hemorrhagic or septic shock, and some of these effects appear to be mediated by estrogen receptors. However, the influence of resveratrol on liver function and hepatic microcirculation after hemorrhagic shock is unknown, and a presumed mediation via estrogen receptors has not been investigated in this context.

**Methods:**

Male Sprague-Dawley rats (200-300g, n = 14/group) underwent hemorrhagic shock for 90 min (MAP 35±5 mmHg) and were resuscitated with shed blood and Ringer’s solution. Animals were treated intravenously with vehicle (1% EtOH), resveratrol (0.2 mg/kg), the unselective estrogen receptor antagonist ICI 182,780 (0.05 mg/kg) or resveratrol + ICI 182,780 prior to retransfusion. Sham-operated animals did not undergo hemorrhage but were treated likewise. After 2 hours of reperfusion, liver function was assessed either by plasma disappearance rate of indocyanine green (PDR_ICG_) or evaluation of hepatic perfusion and hepatic integrity by intravital microscopy, serum enzyme as well as cytokine levels.

**Results:**

Compared to vehicle controls, administration of resveratrol significantly improved PDR_ICG_, hepatic perfusion index and hepatic integrity after hemorrhagic shock. The co-administration of ICI 182,780 completely abolished the protective effect only with regard to liver function.

**Conclusions:**

This study shows that resveratrol may improve liver function and hepatocellular integrity after hemorrhagic shock in rats; estrogen receptors mediate these effects at least partially.

## Introduction

Hemorrhagic shock may result in profound ischemia and reperfusion injury. A traumatic blood loss, for example, can lead to a subsequent inflammatory response, which may eventually result in severe organ dysfunction or complete organ failure. In addition to the involvement of brain, heart and lungs, hepatic injury is likely to occur, as the liver is highly sensitive to the inflammatory processes following ischemia and reperfusion [1].

For organ protection, various strategies have been proposed to reduce reperfusion injury and to mitigate organ damage. Pretreatment or treatment with various antioxidant substances such as melatonin [2–4], traditional herbal medicines [5] or white tea polyphenols [6] may effectively reduce hepatocellular damage or hepatic dysfunction after various types of injury. One of the newer substances with antioxidant properties and a relevant hepatoprotective potential appears to be resveratrol [7].

Resveratrol is a polyphenol found in red grapes and wine and has potent antioxidant properties with potential beneficial effects on various diseases [8]. This polyphenol appears to have an anti-obesity effect [9], may act as an antioxidant against cancer cells [10] and has a positive influence on liver steatosis [11, 12] and cirrhosis [13]. Resveratrol has been shown to be highly effective in reducing reperfusion injury, e.g., after hemorrhagic shock in the liver and heart [14–18]. The mechanism of action of resveratrol appears to involve estrogen receptors [19] and the hepatoprotective effect of resveratrol has been shown to be based on the activation of estrogen receptors [20]. However, little to nothing is known about the influence of resveratrol on *in vivo* liver function and hepatic perfusion. Therefore, this study used a rat model of hemorrhagic shock and subsequent reperfusion to investigate whether resveratrol could affect *in vivo* liver function, as measured by plasma disappearance rate of indocyanine green (PDR_ICG_), or hepatic microcirculation and hepatocellular damage as measured by intravital microscopy.

## Materials and methods

### Drugs and chemicals

All chemicals were obtained from Sigma (Sigma-Aldrich, Munich, Germany) unless stated otherwise. Resveratrol was dissolved in ethanol (EtOH) stock solution and diluted with sterile destilled water to a concentration of 80 μg/ml. ICI 182,780 (Tocris, Bristol, UK) was also dissolved in EtOH stock solution and diluted with sterile destilled water to a concentration of 20 μg/ml; EtOH did not exceed 1.0% in the final preparation.

### Animals

All experiments were performed after approval by the animal experiment committee (Landesamt für Gesundheit und Verbraucherschutz des Saarlandes; Saarbrücken; Germany; approval number 47/2011) and in accordance with the German Animal Welfare Act and ARRIVE guidelines. Male Sprague Dawley rats (200–300 g body weight) were purchased from Charles River (Sulzfeld, Germany). The animals always had free access to water, but pellet feed was withheld for 12 hours prior to surgery. The animals were exposed to a regular light-dark cycle of 12:12 hours; all experiments were started at zeitgeber time (ZT) 02 hours.

### Surgical procedure

The surgical procedures were performed as described above [2, 4]. For sedation, the animals received sevoflurane by inhalation and 60 mg/kg sodium pentobarbital intraperitoneally. The surgical tracheostomy was performed with a polythene tube (SIMS Portex Ltd., UK) to facilitate spontaneous breathing. Both right jugular vein and left carotid artery were equipped with fluid-filled polythene catheters (Smith Medical Int. Ltd., Kent, United Kingdom) for the administration of fluids or drugs and for invasive blood pressure monitoring (Modul 66S, Hewlett Packard, Palo Alto, California). A pressure controlled hemorrhagic shock (MAP 35 ± 5 mmHg for 90 minutes) was induced by rapid withdrawal of arterial blood. 0.2 ml of heparinized blood was used for blood gas analysis (pHOX, Nova Biomedical, Rodermark, Germany) before and after the hemorrhagic shock and at the end of reperfusion (120 minutes). The reperfusion started with the re-transfusion of 60% of the previously withdrawn blood within 5 minutes, followed by a 120 minute reperfusion with acetated Ringer´s solution (volume therapy: 200% of initially withdrawn blood volume in the first hour, 100% in the second hour). After reperfusion, the animals were subjected to either PDR_ICG_ measurement or intravital microscopy.

### Experimental protocols

**Fig 1:**
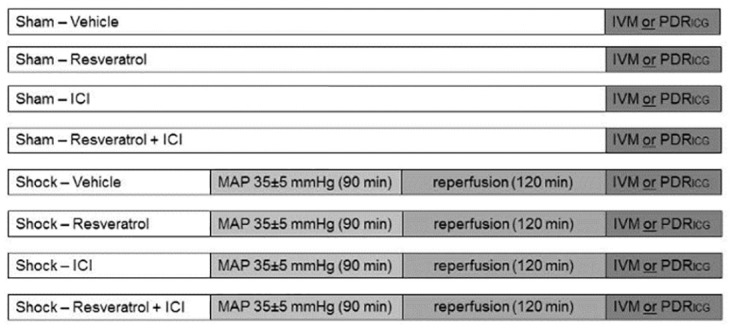
Experimental protocol. For each group, sample size was n = 14; each group was split in n = 7 for evaluation of intravital microscopy (IVM) or plasma disappearance rate of indocyanine green (PDR_ICG_), as both measurements cannot be performed in the same animal; MAP = mean arterial pressure. MAP = mean arterial pressure; ICI = estrogen receptor antagonist ICI 182,780.

Fig 1 gives an overview of the test protocol. Sham-operated animals did not undergo hemorrhage but were infused with 10 ml/kg/h Ringer’s solution and received vehicle EtOH (sham/vehicle), resveratrol 0.2 mg/kg (sham/res), estrogen receptor antagonist ICI 182,780 at 0.05 mg/kg bodyweight 5 min prior to 0.2 mg/kg resveratrol (sham/RES+ICI) or ICI 182,780 alone (sham/ICI) (n = 14 for each group). Shock groups were treated likewise and received EtOH solution (shock/vehicle), resveratrol (shock/RES), resveratrol plus ICI (shock/RES+ICI) or ICI 182,780 alone (shock/ICI) after 90 min of hemorrhagic shock, prior to retransfusion (n = 14 for each group). Finally, the animals were subjected to either PDRICG or intravital microscopy, as neither techniques can be performed on the same animal, resulting in n = 7 for each test.

### Intravital microscopy of the liver

Hepatic intravital microscopy (IVM) was performed as described above [2, 3]. Using a Zeiss Axiotech fluorescence microscope (Axiotech Vario 135, Carl Zeiss Inc., Germany), all microscopic images were projected onto a high-resolution digital video camera with charge-coupled device (C 4742–95, Hammamatsu Photonics, Japan). The rats were subjected to a transverse laparotomy, hepatic ligaments were dissected, and the animals were placed on their left side to expose the left liver lobe on a glass slide. The autofluorescence of Nicotinamide adenine dinucleotide phosphate (NADPH) was evaluated using a 5x objective lens and a filter set with 365 nm excitation and 420 nm emission bandpass, recording 5 visual fields with standardized gain, black level, and amplification settings. NADPH autofluorescence was densisometrically evaluated and expressed as average intensity per hepatic acinus (arbitrary Units [aU]). After intravenous administration of 0.1 mg/kg fluorescein sodium and 0.3 ml of fluorescein isothiocyanate-labelled red blood cells (FITC-RBCs), hepatic microcirculation was determined using a 40x objective lens and a filter set with 485 nm excitation and 520 nm emission bandpass. For each animal 5 video sequences (10 seconds each) were recorded and evaluated as described below. FITC-RBCs were prepared as described above [2, 3]. The hepatocellular injury was assessed 10 minutes after injection of 0.5 mg/kg propidium iodide (PI) using a 40x objective lens, exposure with a 510–560 nm excitation filter and emission bandpass > 590 nm. The PI-labeled nuclei were determined in 5 fields of view in each animal.

### Assessment of hepatic microcirculation

As described above [2, 3] the hepatic microcirculation was analyzed offline with a high-resolution software (SimplePCI, Version 5.3, Compix Inc., USA) by a researcher blinded to the treatment. In each video sequence the perfused sinusoids per 200 μm (SP), sinusoidal diameters (SD) and red blood cell velocity (VRBC) were measured.

### Evaluation of PDR_ICG_

Indocyanine green was infused with 2.5 ml/h for 60 minutes during the second hour of reperfusion to achieve a steady-state as described above [2, 3]. During the ICG infusion, the reperfusion rate of Ringer’s solution was adjusted to a constant total volume. The syringe, lines and tubes were covered with tin foil to prevent photodegradation of ICG. After discontinuation of ICG perfusion, blood was drawn from the arterial line (0.3 ml) after 0, 2, 4, 6, 8, 10, 15 and 20 minutes. The blood was centrifuged at 5,000 g for 10 minutes and the ICG absorption of the plasma was determined spectrophotometrically at 800 nm. The measured absorbance was converted into the corresponding plasma concentration and expressed as a percentage decrease per minute (%/min).

### Quantitative determination of serum enzyme levels

L-Alanine aminotransferase (ALAT), L-aspartate aminotransferase (ASAT), and glutamate dehydrogenase (GLDH) were analyzed with commercially available kits (Roche Diagnostics, Berlin, Germany).

### Blood sample preparation

For evaluation of immune response, each 1.0 mL of whole blood was cultured in sterile polypropylene tubes (Falcon; Becton Dickinson, Lincoln Park, NY) and incubated in a humidified atmosphere with 5.0% CO2 at 37°C for 24 hours, without stimulation (unstimulated immune response) as described previously (Mathes et al Crit Care Med 2014). Twenty-four hours after the onset of culture, the plasma was separated, immediately frozen at –80°C and stored for subsequent analysis of tumor necrosis factor-α (TNF-α), interleukin (IL)-6, and IL-10.

### Cytokine assay

Cytokine concentrations were measured by means of ELISA (OptEIA ELISA, BD Biosciences, Heidelberg, Germany) according to the manufacturer´s instructions, using commercially available kits (Roche Molecular Diagnostics, Mannheim, Germany). Aliquots of supernatants were thawed at room temperature, positive controls of each cytokine were measured routinely with each assay. The calculated interassay and intraassay coefficients of variance were 4.2% and 3.7% for TNF-α, 5.9% and 4.8% for IL-6, as well as 5.4% and 3.9% for IL-10. The minimal detectable concentrations, as estimated from the average optical density reading of zero standards plus 2 SDs, were 2.6 pg/mL for TNF-α, 1.8 pg/mL for IL-6, and 1.0 pg/mL for IL-10.

### Statistical analysis

Unless stated otherwise, the data are given as mean value ± standard deviation. The statistical analysis was performed with Sigma Plot 12.0 (SystatSoftware, Erkrath, Germany). The data were tested for normal distribution (Kolomogorov-Smirnov test) and analyzed using one-way analysis of variance (ANOVA), followed by post-hoc multiple comparisons with the Student-Newman-Keuls test. A p-value of less than 0.05 was considered significant for all statistical analyses.

## Results

### Macrohemodynamic parameters and blood gas analysis

**Fig 2:**
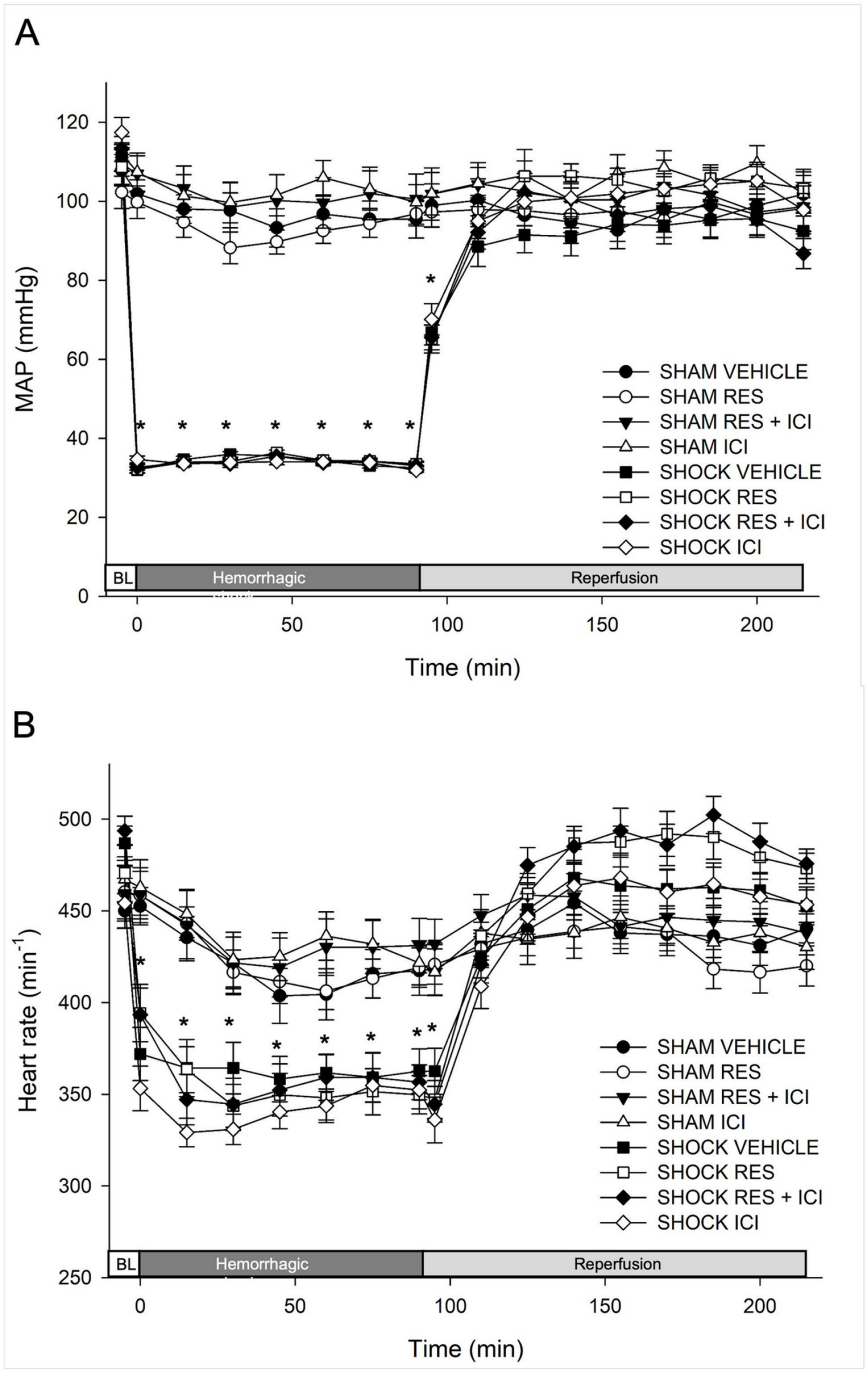
Hemodynamic parameters. (a) Mean arterial pressure (MAP) remained stable in sham-operated animals and was returned to normal after retransfusion in shock animals. An Asterisk (*) indicates a significant difference between all shock and all sham groups; (b) There was a typical decrease in heart rate in all shock animals. All changes returned to normal after retransfusion. An Asterisk (*) indicates a significant difference between all shock and all sham groups. Res = Resveratrol; ICI = estrogen receptor antagonist ICI 182,780.

**Fig 3:**
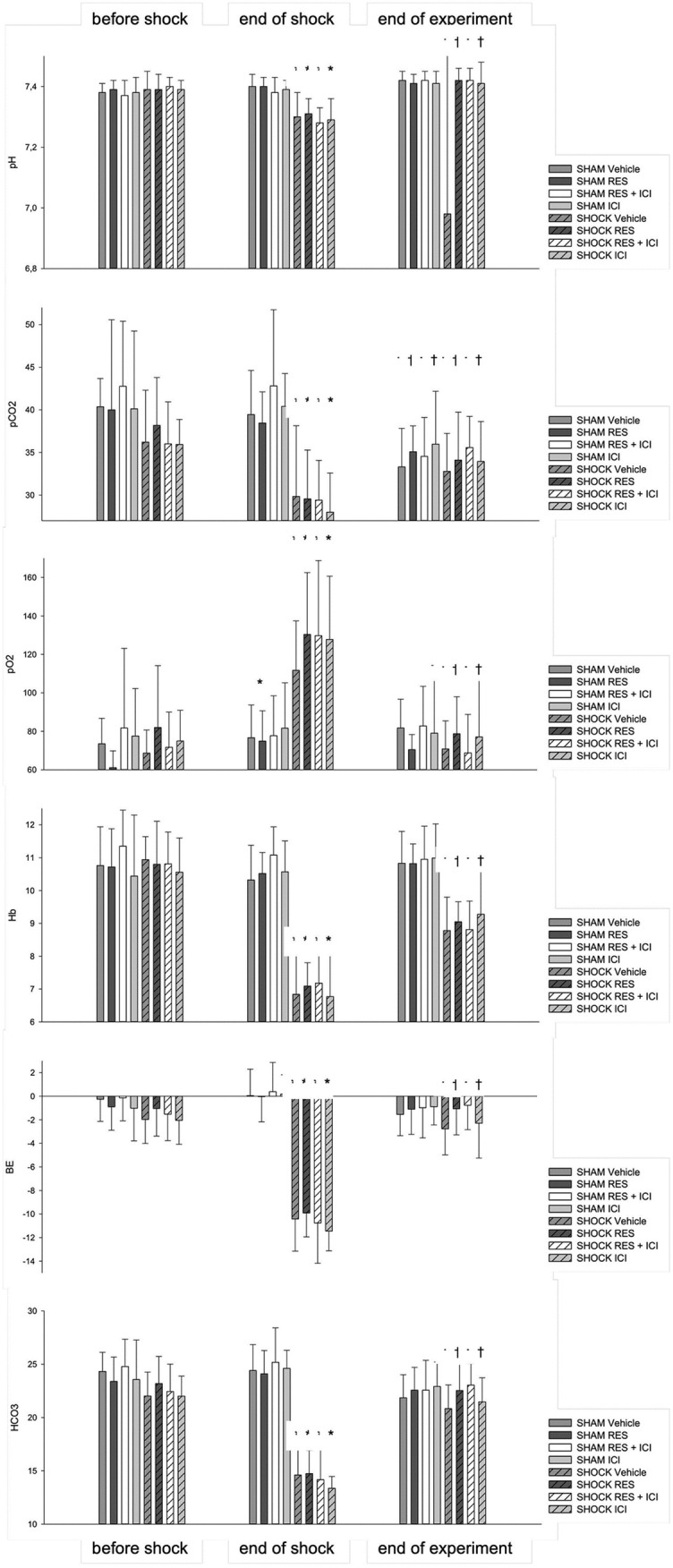
Blood gas values. An asterisk (*) indicates p < 0.05 vs. baseline; a cross (†) indicates p < 0.05 vs. values at 90 min. Data are expressed as mean ± SD (n = 14). Res = Resveratrol; ICI = estrogen receptor antagonist ICI 182,780; Hb = hemoglobin; BE = base excess.

All animals presented with comparable baseline values for heart rate and MAP (Fig 2) as well as for respiratory parameters, acid base balance and hemoglobin content (Fig 3). Resveratrol, ICI or the combination of both did not significantly alter any parameter in the sham controls. Hemorrhagic shock resulted in a typical decrease in heart rate in all groups. After hemorrhagic shock, all animals showed a significant decrease in hemoglobin and base excess levels (p < 0.05 vs. baseline); this was reversible in all groups after retransfusion (p < 0.05 vs. end of study). The blood volume withdrawn, adjusted to body weight, did not differ significantly between the shock groups (Vehicle group 42.62 (±6.49) mL/kg bodyweight, Resveratrol 43.43 (±4.74) mL/kg, Resveratrol+ICI 42.31 (±2.78) mL/kg, and ICI 42.96 (±4.54) mL/kg).

### Plasma disappearance rate of indocyanine green

**Fig 4:**
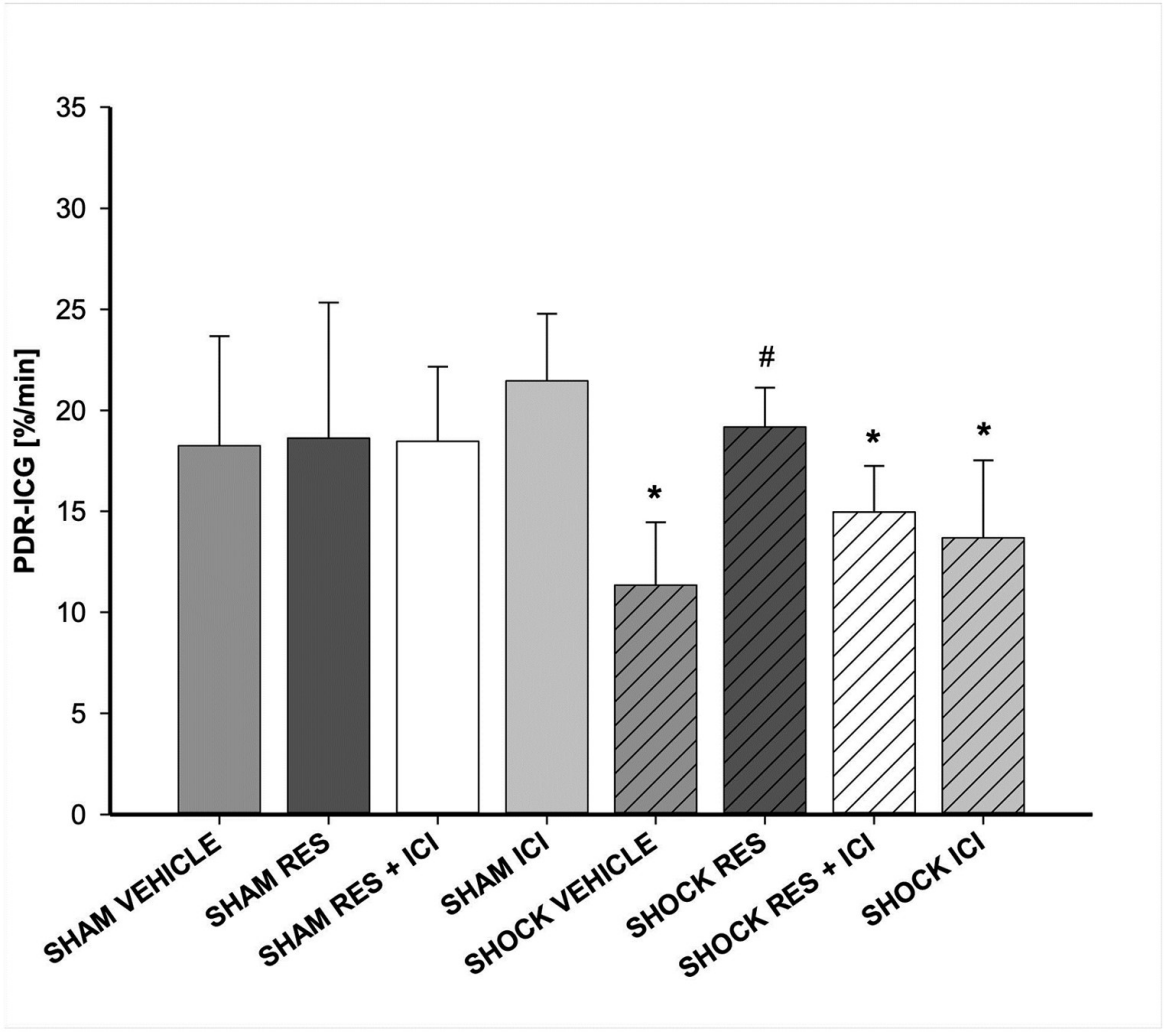
Liver function measured by PDR_ICG_. Plasma disappearance rate of indocyanine green (PDR_ICG_) as a marker of liver function was significantly reduced in shock animals, irrespective of ICI treatment. Therapy with resveratrol (RES) attenuated this effect significantly; estrogen receptor antagonist ICI abolished the protective effect of resveratrol. n = 7 per group; an asterisk (*) indicates p < 0.05 vs. all sham groups; a pound sign (#) indicates p < 0.05 vs. all other shock groups.

Regardless of resveratrol or ICI treatment, sham-operated animals presented normal PDR_ICG_ levels (Fig 4). Induction of hemorrhagic shock resulted in a significant decrease of PDR_ICG_ levels compared to sham controls (p < 0.05). Administration of resveratrol resulted in a significant improvement in liver function compared to vehicle controls (p < 0.05). ICI completely eliminated this protective effect (p < 0.05 vs. shock/resveratrol).

### NAD(P)H autofluorescence

**Fig 5:**
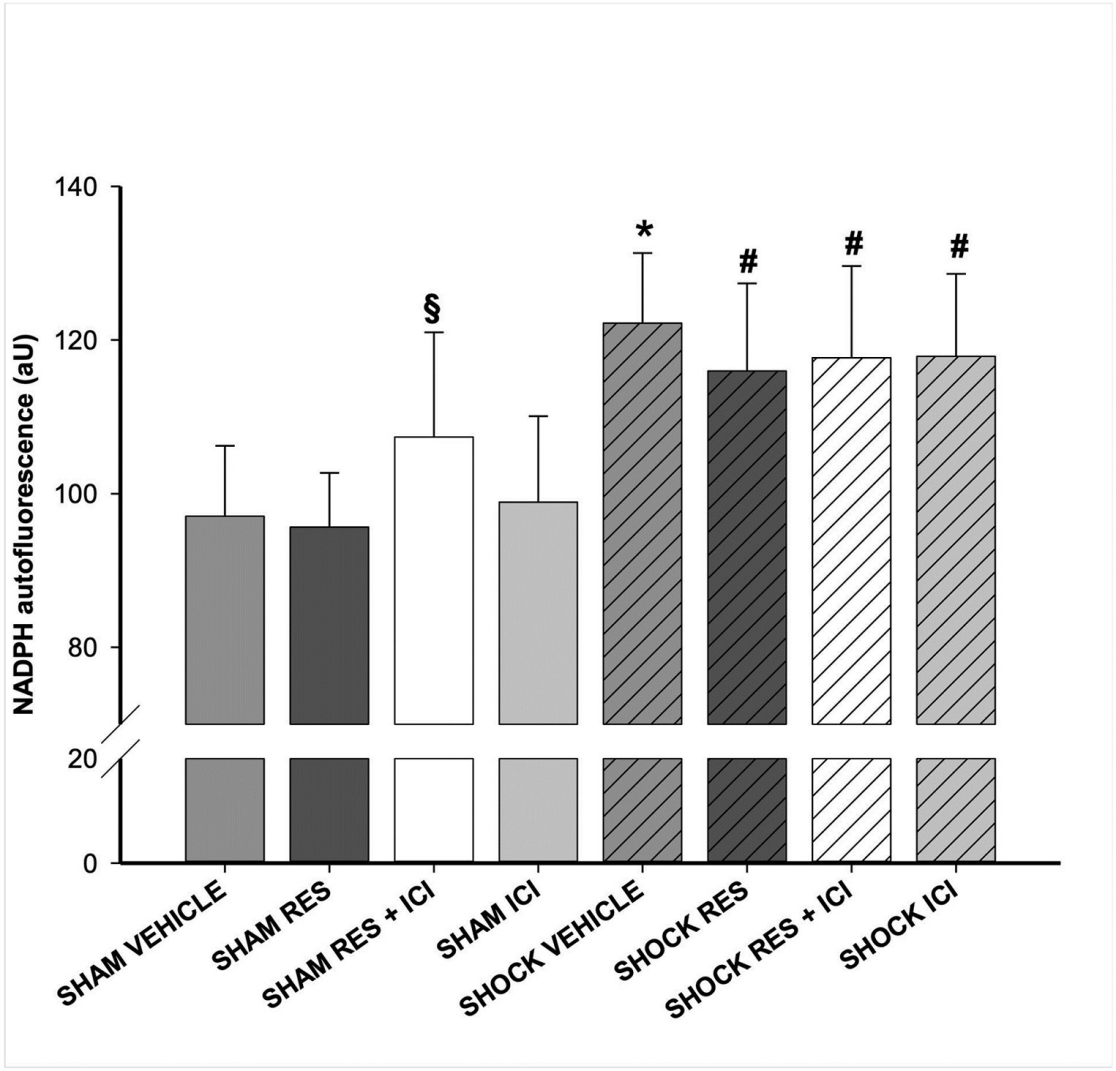
Hepatic redox state measured by NAD(P)H autofluorescence. Nicotinamide adenine dinucleotide phosphate (reduced form) (NADPH) auto fluorescence as a marker of hepatic redox state. n = 7 per group; an asterisk (*) indicates p < 0.05 vs. all sham groups; a paragraph sign (§) indicates p < 0.05 vs. all other sham groups, and a pound sign (#) indicates p < 0.05 vs. sham/vehicle, sham/resveratrol (RES) or sham/ICI.

**Fig 6:**
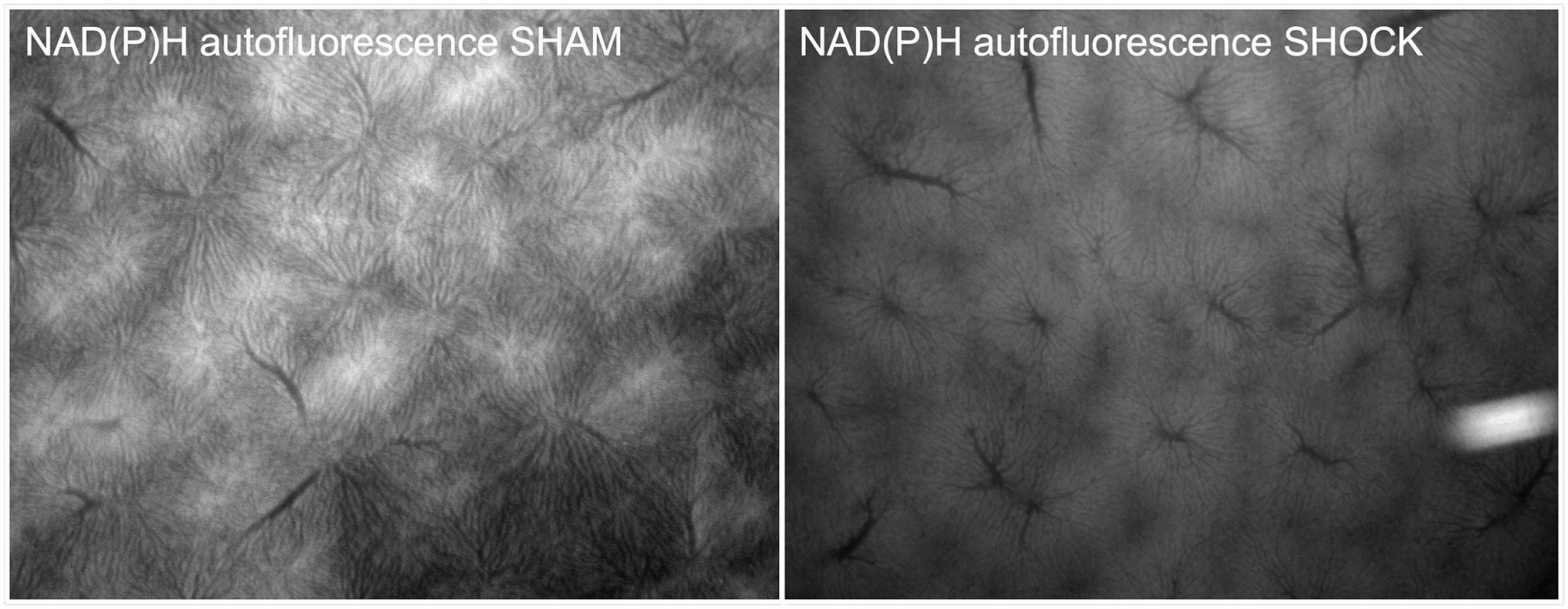
NAD(P)H autofluorescence for SHAM vehicle and SHOCK vehicle.

The administration of resveratrol resulted in normal NADPH autofluorescence in sham-operated animals, but resveratrol plus ICI resulted in significantly increased NADPH autofluorescence even in sham controls (Fig 5). Compared to sham operated animals, NADPH autofluorescence was significantly increased in the vehicle group after hemorrhagic shock (p < 0.05). This effect was not attenuated by the administration of resveratrol. Simultaneous administration of ICI had no significant effect on NADPH autofluorescence. Fig 6 illustrates the NAD(P)H autofluorescence as an example.

### Hepatic microcirculation

**Fig 7:**
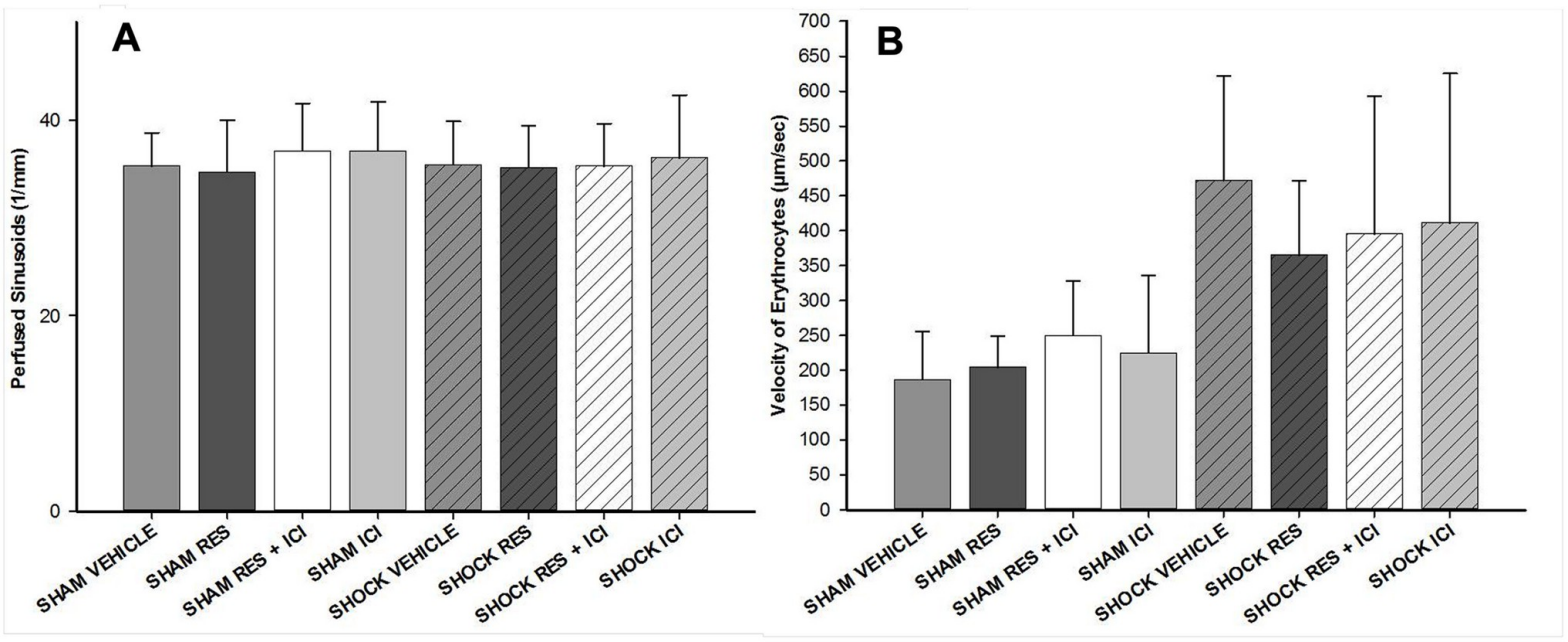
Analysis of hepatic microcirculation. Neither the number of perfused sinusoids 7a nor velocity of erythrocytes 7b changed significantly between any group, irrespective of the treatment. RES = resveratrol. n = 7 per group.

The number of perfused sinusoids per millimeter did not differ significantly between the groups, nor did the velocity of the erythrocytes (Fig 7). A trend towards higher erythrocyte velocity in animals after haemorrhagic shock was observed. However, this was not statistically significant. As a result, hepatic volumetric blood flow and hepatic perfusion index were not calculated.

### Hepatocellular injury: PI-stained cells

**Fig 8:**
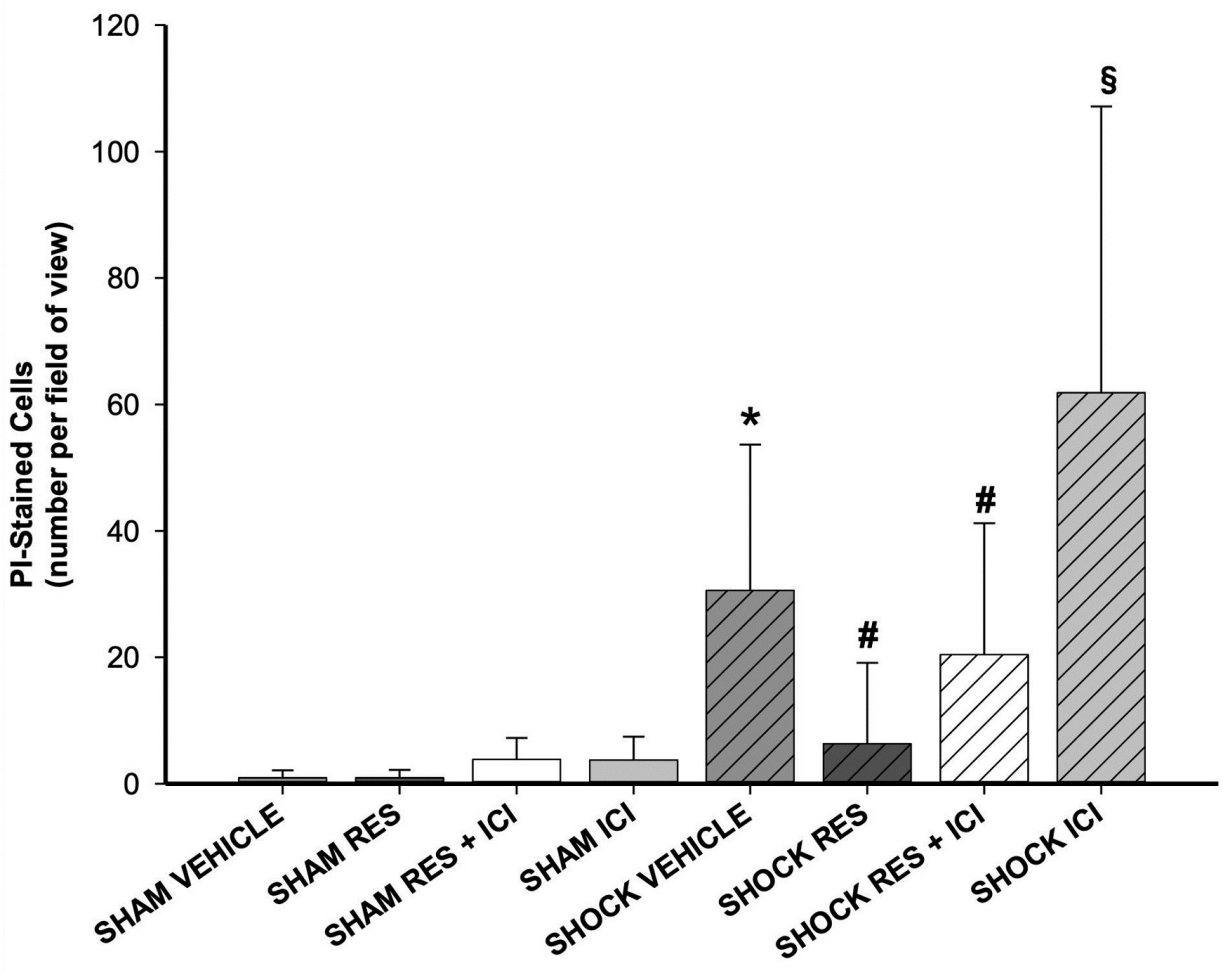
Hepatic injury assessed by PI-stained cells. Hepatic injury assessed by propidium-iodide staining of cells was significantly higher in all shock groups except resveratrol (RES) treated animals, compared to sham controls. An asterisk (*) indicates p < 0.05 vs. sham groups. n = 7 per group.

**Fig 9:**
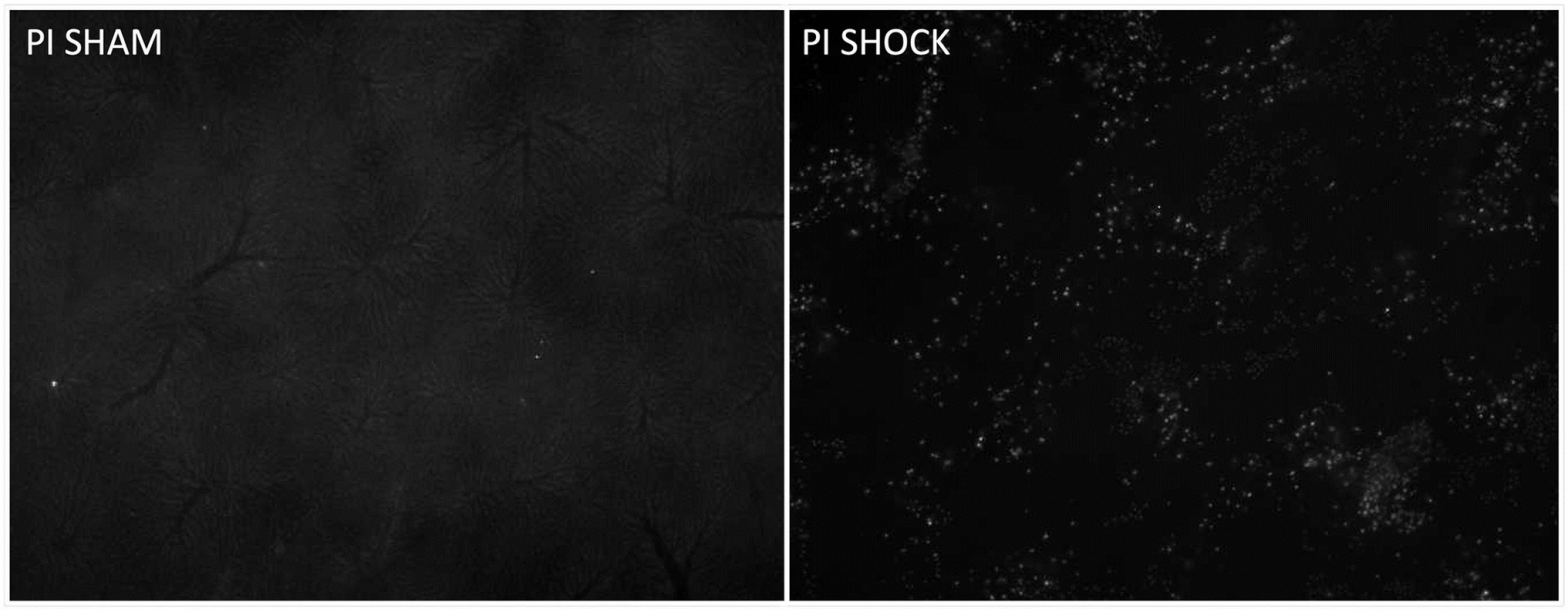
Illustrates exemplary cell damage using propidium Iodide labeled nuclei (luminous dots). The image from the SHAM group shows almost no cell damage, whereas the SHOCK vehicle group shows massive cell damage.

Regardless of resveratrol or ICI treatment, sham operated animals showed no significant signs of hepatocellular damage, which was assessed using PI-stained cells (Fig 8). Induction of hemorrhagic shock resulted in a significant increase in PI-stained cells in vehicle, resveratrol plus ICI and ICI-treated groups (p < 0.05 vs. sham). This effect was attenuated by resveratrol and showed no significant difference to the sham groups. However, the difference between shock/vehicle and shock/resveratrol groups was not statistically significant. Fig 9 demonstrates exemplary the intravital microscopy of vehicle treated SHAM and SHOCK liver.

### Hepatocellular injury: Serum enzyme and cytokine levels

**Fig 10:**
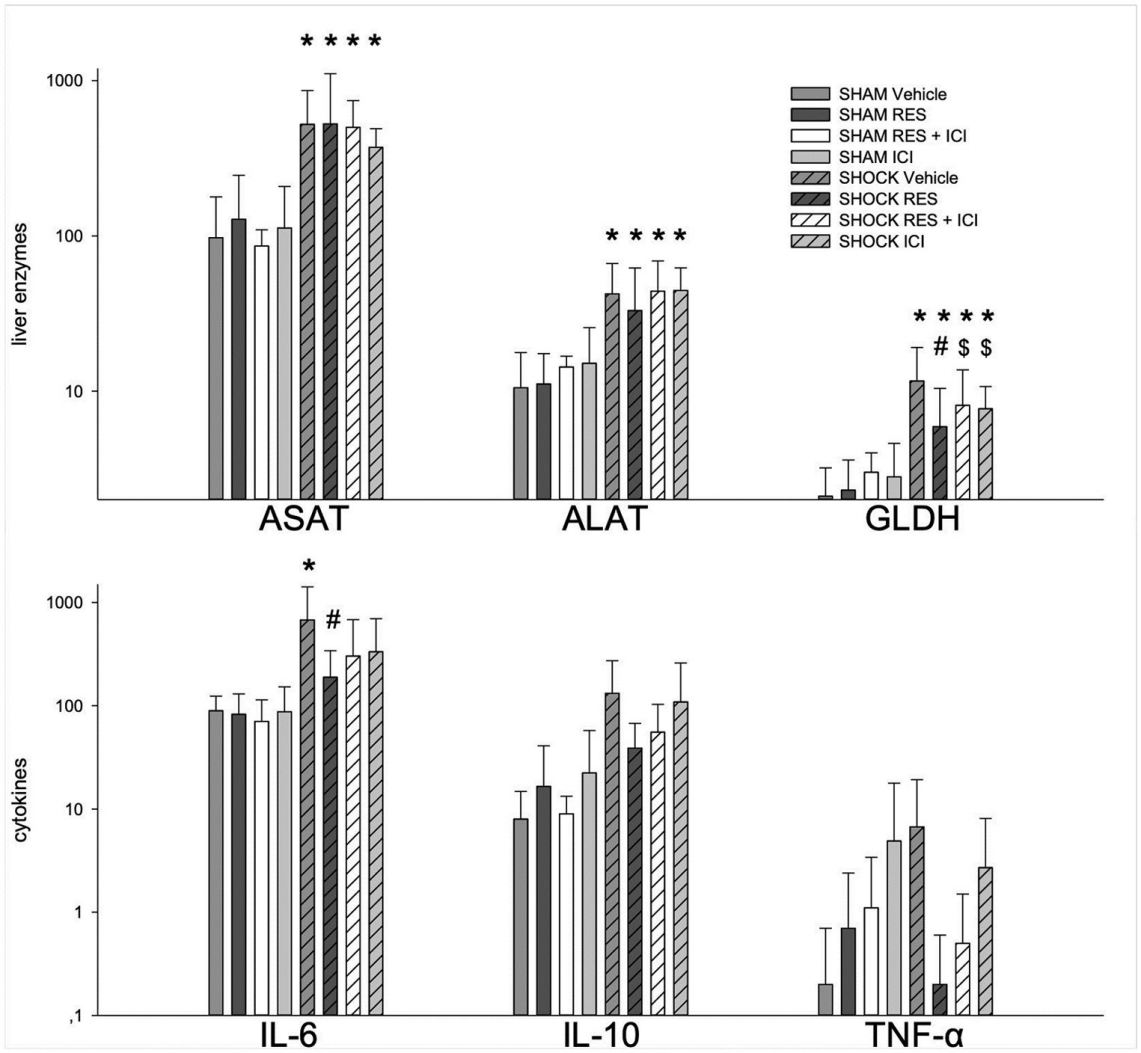
Hepatic injury assessed by liver enzymes and serum cytokines. An asterisk (*) indicates p < 0.05 vs. All SHAM; a pound sign (#) indicates p < 0.05 vs SHOCK Vehicle; a dollar sign ($) indicates p < 0.05 vs. SHOCK Resveratrol Shock/Resveratrol. n = 14 per group.

Sham-operated animals showed no significant signs of hepatocellular damage when assessed using serum liver enzymes, irrespective of resveratrol or ICI treatment (Fig 10). Induction of hemorrhagic shock resulted in a significant increase of ASAT, ALAT and GLDH in all groups (p < 0.05 vs. sham). Resveratrol did not significantly alter this increase, except for GLDH. The latter effect was significantly reversed by ICI. Sham-operated animals showed comparable cytokine levels. Only shock vehicle showed statistically elevated IL-6 levels compared to all SHAM groups. Resveratrol treated animals with SHOCK showed significant reduced IL-6 levels compared to vehicle treated SHOCK animals.

## Discussion

This study shows that intravenous therapy with resveratrol may improve both liver function measured *in vivo* by PDR_ICG_ and hepatocellular injury assessed with PI-stained cells after hemorrhagic shock in rats. We observed no significant differences in the effect of resveratrol on hepatic redox state or intravital hepatic microcirculation. The beneficial effects of resveratrol in relation to PDR_ICG_ could be antagonized using the estrogen receptor antagonist ICI 182,780.

Ischemia and reperfusion (I/R) injury, as it occurs after hemorrhagic shock, leads to oxidative stress, and naturally occurring polyphenol resveratrol may play a key role in modifying I/R injury and the associated pathways. After lipopolysaccharide (LPS) induced oxidative stress, resveratrol was able to counteract LPS-induced antioxidant enzyme depletion of superoxide dismutase (SOD) and catalase (CAT), as well as iron sequestration from rat liver [21]. Similar results for SOD, CAT, glutathione peroxidase (GPx) and hepatocellular injury were found after resveratrol treatment of heat stress induced hepatotoxicity [22]. In this regard, the effect of resveratrol on SOD, CAT and GPx appears to be mediated by an influence on the expression of the hepatic heat shock protein (HSP) 70 and 90 in quail [23]. In heat-stressed rats, oral treatment with resveratrol could also modify SOD, GPx, HSP-70, tumor necrosis factor alpha (TNF-α), toll-like receptor 4 and interleukin 10 in the liver [24, 25]. In the cell culture of HepG2 cells, resveratrol induced free fatty acid or carbon tetrachloride apoptosis by reducing stress to the endoplasmatic reticulum [26].

While these results impressively demonstrate the antioxidative and hepatoprotective potential of resveratrol, most of these studies were performed using static tests to assess the liver. Nevertheless, liver function can be assessed by static and dynamic tests, and the use of dynamic tests is highly relevant to gain insight into otherwise discrete but highly relevant hepatocellular effects, such as excretory capacity.

Therefore, in this study we have used a combination of dynamic *in vivo* tests and static tests to evaluate liver function. Regarding liver function as measured by PDR_ICG_, resveratrol therapy was able to restore excretory liver function to near normal levels after hemorrhagic shock, while hemorrhagic shock alone had adverse effects of PDR_ICG_. The use of the estrogen receptor antagonist ICI 182,780 removed this protective effect, suggesting that the hepatoprotective potential of resveratrol regarding PDR_ICG_ could be mediated by estrogen receptors. To our knowledge, there are two further investigations on the effect of resveratrol in shock via ER on the liver [20, 27]. In these publications, ICI 182,780 was also used to block signal transduction via ER. One study focused on endothelial dysfunction of the aorta at the mRNA level in a rat model [27]. Additional findings included ASAT and ALAT, which were attenuated in the setting of resveratrol therapy; ICI 182,780 reversed this effect. The second work focused on inflammation and immune effects in the liver, in which liver tissue was processed by protein assays [20]. Here resveratrol was able to attenuate inflammation, which was reversed by simultaneous blockade with ICI 182,780.

While our study complements the previous findings with static methods, it is also the first investigation showing the positive effect of resveratrol on liver function in an *in vivo* model with both static and dynamic tests, using PDR_ICG_ for excretory liver function and PI-staining of hepatic injury Administration of ICI 182,780 and consecutive blockade of ER attenuated the resveratrol effect. Therefore, this is the first study to demonstrate that resveratrol has an immediate therapeutic mechanism of action via estrogen receptors in the rat liver, with positive influence on cell damage and organ function in an *in vivo* model of hemorrhagic shock and resuscitation.

Two alternative mechanisms of resveratrol, which have been investigated in liver ischemia or shock, are mediated by Sirtuin 1 (SIRT-1) and adenosine mono phosphate activated protein kinase (AMPK). Powell et al. found in vivo and vitro prevention of liver cell death in hemorrhagic shock and hypoxic injury, respectively, by possible SIRT-1 modulation of p53 and NF- κB [17]. Li et al. found an increased upregulation and activation of SIRT-1 in hemorrhagic shock [28]. AMPK appears to have protective effects in ischemia-reperfusion injury of the liver [29]. There is some evidence that resveratrol activates AMPK in a dose-dependent manner directly or via SIRT-1 [30].

Interestingly, in our experiment, the hemorrhage induced attenuation of the hepatic redox state, measured by NAD(P)H autofluorescence, was not significantly influenced by resveratrol. Although we observed a discrete optical difference in the NAD(P)H autofluorescene of shocked animals after treatment with resveratrol, it was not statistically significant. Resveratrol is known as an antioxidant. However, this antioxidative effect seems to be dose dependent. Hassan-Khabbar et al. investigated the antioxidant properties alia of trans-resveratrol in a selective in vivo model of hepatic ischemia reperfusion injury [31]. Antioxidative effects were found at low doses of trans-Resveratrol, which decreased at higher doses. Other studies in rodents showed the antioxidative potential even at high doses [18, 28]. Despite the direct antioxidative potential of resveratrol, the antioxidative effect appears to be mediated mainly by SIRT-1 through the restoration of mitochondria and the attenuation of ROS production [18, 32]. The hepatoprotective effects we have identified appear to be caused by estrogen receptors, since the estrogen receptor antagonist ICI 182,780 reduces these identified effects. We have not investigated the effects of SIRT-1 and the underlying mechanisms, so we cannot draw further conclusions as to why we have not observed any significant effects of resveratrol on NAD(P)H.

In addition, the shock-impaired hepatic microcirculation, as assessed by intravital microscopy, was not significantly altered by resveratrol treatment. It is not an unusual observation that the number of perfused sinusoids is not always altered by hemorrhagic shock (see [2]). However, in this study the erythrocyte velocity was increased after hemorrhagic shock without reaching a significant level. Although it would be possible to calculate volumetric blood flow and hepatic perfusion index from these values, and although these calculations could–theoretically–lead to statistically significant differences, the authors believe that this would not be scientifically correct. The “raw” parameters were not altered, and artificial parameters based solely on calculations should not mask the fact that these results for hepatic microcirculation were not as expected. Therefore, hepatic perfusion index is not shown. Descriptively, sinusoidal width was smaller in animals after hemorrhagic shock, especially after treatment with ICI 182,780. The hepatic erythrocyte velocity was generally lower in sham animals and higher in shock animals.

Hepatocellular integrity was evaluated by PI staining and serum liver enzyme analysis. Here, hemorrhagic shock resulted in significantly increased levels of serum ASAT, ALAT and GLDH as well as PI-stained cells, the latter assessed by intravital microscopy. Treatment with resveratrol was able to attenuate these effects for PI-stained cells and for GLDH. Co-administration of ICI 182,780 abolished the beneficial effect of resveratrol. It is a typical finding that serum liver enzymes evaluated by static laboratory methods do not always correspond with all aspects of hepatic integrity, as observed *in vivo* with intravital microscopy [33]; this has been discussed previously [33–35]. For example, although GLDH appears to be one of the most effective biomarkers for hepatic injury in rat, it shows its peak only 18 h after liver injury. In this study however, we evaluated serum liver enzymes 4 h after hemorrhage and resuscitation. It is reasonable to assume that pronounced differences could be registered after a longer time. Yet, previous investigations from our laboratory show that the selected time frame is a good compromise for evaluation of different parameters of liver injury and reflects a clinically relevant period in which putative therapeutic influences would still be of interest.

ALAT and GLDH represent the more liver specific enzymes, whereby ASAT can also represent myocardial, skeletal muscle, brain or kidney injury. And so, an unspecific raise of ASAT even in sham animals due to laparotomy appears to be possible. In our investigation there is the possibility of an elevated ASAT value due to surgical manipulation in all groups. However, this effect does not play a crucial role, as all animals underwent a comparable surgical trauma by laparotomy. And further the ASAT enzyme level is consistent within the SHAM group. The absolute level of serum amino transaminases is low compared to Yu and co-workers which is because of the timing of serum enzyme level assessment [20]. We assessed the serum transaminases two hours after hemorrhagic shock and reperfusion and Yu assessed after 24 hours with a greater likelihood of mapping the peak. Yu was also able to attenuate the serum amino transaminases significantly with the administration of resveratrol.

Thus, our study clearly shows that resveratrol therapy after hemorrhagic shock can improve liver function and reduce hepatocellular damage in rats. These effects appear to be mediated by the activation of estrogen receptors and are independent of relevant influences on hepatic microcirculation. Similar results were obtained by Yu et al., who were also able to show that the hepatoprotective effects of resveratrol are mediated by an estrogen-receptor-dependent upregulation of a pathway involving p38 mitogen-activated protein kinase and heme oxygenase 1 [20, 36]. Further targets of resveratrol signaling include the sirtuin 1 superoxide dismutase 2 pathway (SIRT1-SOD2) [28, 36] as well as the SIRT1-mediated high mobility group protein box 1 nucleocytoplasmatic translocation pathway [37].

Our study is limited in various extents, some of which have already been discussed above. One relevant limitation is that our results on hepatic microcirculation did not meet all criteria we usually achieve in previous studies. Nevertheless, intravital microscopy of rat liver is a challenging investigation, and our research group has more than two decades experience with this technique: The chosen group size was usually sufficient to show significant differences in the past. Ultimately, it cannot be said with certainty why the expected results in hepatic microcirculation did not materialize, although we used a standardized protocol. Resveratrol itself has an effect on endothelial nitric oxide synthase (eNOS) and may cause vascular dilatation and that may influence perfusion, diameter and velocity [38]. However, this also does not satisfactorily explain the result.

In addition to these limitations, our study also has several strengths. First, this study is the first to use in vivo dynamic tests to investigate the hepatoprotective effect of resveratrol.

The PDR_ICG_ is a sensitive measurement for evaluating liver function in shock [39] and intravital microscopy, which provides direct insight into the shock to the liver. Static liver function measurements such as serum liver enzymes increase after 12 to 48 hours and do not allow real-time assessment of liver function [40]. Secondly, this study demonstrated that estrogen receptors mediate not only the beneficial effects of resveratrol on hepatocellular injury, but also on hepatocellular excretory function as measured by PDR_ICG_. Third, our pressure-controlled model of hemorrhagic shock was followed by resveratrol therapy, which is resembling a model that is close to clinical reality. In the past, resveratrol has been used in numerous other rodent models as a pre-treatment or oral supplement. While these studies provide interesting data on fascinating mechanisms, our data allow insight into principles that–in the next step–could be applied at the bedside.

## Conclusions

We would like to conclude that the presented results show that resveratrol therapy after hemorrhagic shock can improve both liver function as measured by PDR_ICG_ and hepatocellular injury as measured by PI-stained cells and GLDH in rats. These effects were antagonized by the estrogen receptor antagonist ICI 182,780, indicating that the protective effects of resveratrol may be mediated by estrogen receptors.
